# Preferences regarding emerging HIV prevention technologies among Toronto men who have sex with men: a discrete choice experiment

**DOI:** 10.1038/s41598-021-01634-3

**Published:** 2021-11-15

**Authors:** Darrell H. S. Tan, Jayoti Rana, Zavare Tengra, Trevor A. Hart, James Wilton, Ahmed M. Bayoumi

**Affiliations:** 1 Division of Infectious Diseases, St. Michael’s Hospital, 30 Bond St., Toronto, ON M5B 1W8 Canada; 2 MAP Centre for Urban Health Solutions, St. Michael’s Hospital, 30 Bond St., Toronto, ON M5B 1W8 Canada; 3 Department of Medicine, University of Toronto, Toronto, Canada; 4 Institute for Health Policy, Management and Evaluation, University of Toronto, Toronto, Canada; 5 Hassle Free Clinic, Toronto, Canada; 6 Department of Psychology, Ryerson University, Toronto, Canada; 7 Dalla Lana School of Public Health, University of Toronto, Toronto, Canada; 8 BC Centre for Disease Control, Vancouver, Canada; 9 Department of Medicine, St. Michael’s Hospital, Toronto, Canada

**Keywords:** HIV infections, Preventive medicine

## Abstract

New forms of HIV pre-exposure prophylaxis (PrEP) include long-acting injectables and topical microbicides, each with unique attributes that may appeal to distinct users. We used a discrete choice experiment to characterize preferences for new PrEP formulations among Toronto men who have sex with men. MSM undergoing anonymous HIV testing completed a discrete choice experiment with 12 choice sets by selecting their preferred option within each set. Each set included “usual methods to prevent HIV” (excluding PrEP) as one alternative and two hypothetical PrEP alternatives, which differed according formulation/dosing, side effects (none/mild), risk of drug resistance (none/low/moderate), and HIV prevention efficacy (50%, 65%, 80% or 99% risk reduction). We used mixed logistic regression to infer preferences for PrEP attributes and calculate the marginal rate of substitution between efficacy and other PrEP attributes. 306 men with median (interquartile range) age = 29 (25, 36) years participated, and reported 6 (3, 10) partners and 0 (0, 2) condomless receptive anal sex acts in the preceding six months. An on-demand pill was the most preferred formulation, followed by a monthly injection, daily pill, and on-demand rectal gel. Drug resistance was an important determinant of preferences if the risk was moderate, but not if it was low. The minimum efficacy required for an on-demand pill to be preferred over no PrEP was 32.6% (95%CI = 21.2–43.9%); for a daily pill, injections, and rectal gel, minimum efficacy was 57.9% (95%CI = 44.1–71.7%), 40.1% (27.0–53.2%), and 71.3% (60.5–82.1%), respectively. Attitudes towards PrEP formulations vary among men who have sex with men, with on-demand pills and monthly injections having the highest average preference scores. Understanding these preferences may help to predict uptake.

## Introduction

Globally, a disproportionate burden of new HIV infections occurs among gay, bisexual and other men who have sex with men (gbMSM). Both daily^1,2^ and on-demand^3,4^ oral tenofovir disoproxil fumarate/emtricitabine (TDF/FTC) are safe, effective and available as pre-exposure prophylaxis (PrEP) among gbMSM, as is daily oral tenofovir alafenamide/emtricitabine (TAF/FTC)^5^. However, additional prevention options might appeal to some individuals. For example, injectable long-acting cabotegravir was recently demonstrated to be superior to daily oral TDF/FTC as PrEP among gbMSM in the HPTN 083 trial^6^. Building on the early success of tenofovir gel as a vaginal microbicide^7^, a reduced-glycerin formulation of tenofovir 1% gel was found to have favourable safety, acceptability and adherence among gbMSM^8,9^, and a combined rectal formulation of tenofovir alafenamide with elvitegravir is under investigation^10^.

As the HIV prevention landscape continues to evolve, individuals may soon have a range of PrEP options, each with different dosing regimens, effectiveness, side effect profiles and other properties. In addition, many health decisions do not represent a simple choice between alternatives; rather, there are often trade-offs which are important to understand. For example, understanding the extent to which people may be willing to sacrifice some degree of HIV protection (such as choosing a less efficacious product) in favour of a more desirable characteristic (such as more convenient route of administration) may inform how clinicians counsel patients and may help policy-makers project uptake, adherence and population-level impact. A discrete choice experiment (DCE) is a survey-based method rooted in health economic and decision sciences theory that aims to quantify such preferences from the consumers’ perspective^11^. We used a DCE to characterize preferences for existing and forthcoming PrEP modalities among gbMSM in Toronto.

## Methods

We embedded a DCE within a larger, paper-based 50-item anonymous survey about awareness of, willingness to use, and uptake of TDF/FTC-based PrEP. Results from the larger survey have been published^12^. Participants were adult, English-speaking cisgender male gbMSM undergoing voluntary anonymous point-of-care HIV testing at a busy sexual health clinic in downtown Toronto, as well as three satellite HIV testing clinics, between May and August 2016. Participants using TDF/FTC-based PrEP were excluded from the DCE analysis. Although the questionnaire was self-administered in private, a study coordinator was nearby to ensure that questions were properly understood.

### DCE design

**Figure 1:**
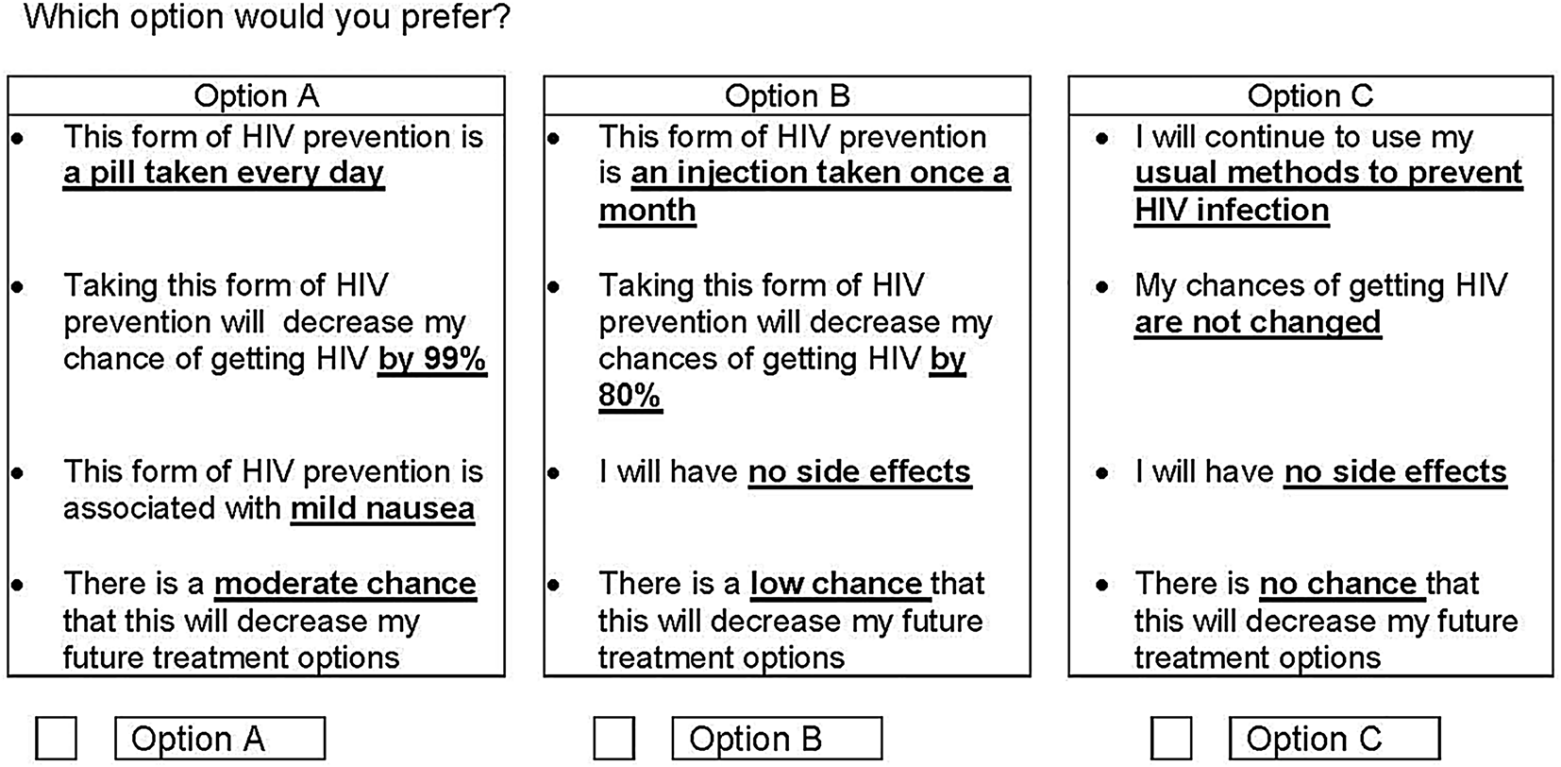
Example DCE choice set. Caption: Participants were presented with 12 such choice sets and selected one option from each set of three. Each choice set varied in terms of the PrEP formulation, the efficacy for HIV prevention, potential for mild side effects, and chance of decreasing future treatment options.

A DCE presents individuals with a series of choice sets and preferences are inferred through analyzing their selections^13–15^. We presented each participant with 12 choice sets, each consisting of two PrEP alternatives and a status quo condition (Fig. 1). Respondents were asked which of the three alternatives they most preferred. The PrEP alternatives were described by a set of attributes, each with a defined number of levels. We used an unlabeled design, in which alternatives were labeled with generic descriptors (A, B, and C) and the combination of attribute levels represented hypothetical, rather than actual, PrEP options.

We selected attributes and levels based on literature regarding PrEP formulations for gbMSM that were in existence or in development at the time of study design (2014–2016) and included four attributes. First, the *route of administration and dosing frequency* was either a daily pill, an on-demand pill (“a pill taken before and for two days after sex”), a monthly injection, or an on-demand rectal gel. Second, *side effects* could either be absent or mild. A single specific side effect was associated with each formulation/administration route (nausea for pills, injection site reaction for injections, rectal discomfort for rectal gels). Third, the *risk of inducing HIV drug resistance* included no risk, a low risk, or a moderate risk of decreasing future HIV treatment options. Finally, *HIV prevention efficacy* was either 50%, 65%, 80% or 99% risk reduction. We assumed that the status quo condition was associated with no side effects, no risk of decreasing future HIV treatment options, and no decrease from baseline in HIV acquisition risk (see “Statistical analysis” section).

Because testing all possible combinations of attribute levels is not feasible, DCEs combine attribute levels using fractional factorial efficient designs^16^. Such designs identify a manageable number of combinations that is unlikely to result in respondent fatigue while facilitating estimation of the effects of interest. We pilot-tested our initial DCE design in 66 individuals for two purposes. First, we assessed whether the DCE wording was clear, whether attributes and levels were appropriate, and whether individuals were willing to make trade-offs between attribute levels. We made minor changes to the wording of the survey, accordingly. Second, we used quantitative results of the pilot as inputs for the final d-efficient survey design.

### Statistical analysis

We excluded participants if they were transgender men (for whom alternative PrEP strategies, such as vaginal solutions and rings may be relevant but were not included in the DCE), if they were taking PrEP already, or if they gave invariant responses (selected the same option in each choice set). We summarized participant characteristics using descriptive statistics. As a check of validity, we examined the association between expressed level of interest in PrEP and the frequency with which respondents selected “Usual Care” (no PrEP) using Pearson’s chi-squared statistic. We also calculated Mc-Fadden’s pseudo R-squared^17^, using the initial log-likelihood value from of a model with starting values based on a model without error components to approximate the log-likelihood for the model without predictors.

We analyzed survey results using a mixed multinomial logit model (also called a random parameter logit model), in which the selected option is the dependent variable, the attribute levels are independent variables, and some coefficients are expressed as random variables, thereby incorporating individual-level heterogeneity in responses^18^. The model requires that at least one parameter be fixed, which indicates preference homogeneity for such parameters. Typically, this parameter also serves as the denominator for marginal rate of substitution calculations. We followed this convention and assumed that the coefficients for the efficacy parameter were fixed. We assumed all other parameters were random and entered each attribute level as an indicator (dummy) variable into the model. In addition to coefficients for each attribute level, the model included a coefficient indicating whether individuals selected the Usual Care option (an alternative-specific constant). We also included terms for error components, which allows the two PrEP alternatives to be correlated with each other and reflects the “nested” nature of the choice (i.e., individuals first chose whether to use PrEP or Usual Care; those who chose PrEP then selected between the two PrEP alternatives). All models were run using 1000 Halton draws (an algorithm for performing maximum likelihood estimation by simulation) and assuming independence of the random coefficients. We also adjusted standard errors to reflect clustering at the individual level.

We initially entered efficacy (risk reduction) as a linear term into the regression model. However, this has two disadvantages. First, many individuals consider probabilities non-linearly (i.e., they have different preferences for a 10% risk reduction from 95 to 85% versus from 50 to 40%). Second, entering probabilities as linear terms does not constrain the range of possible results, which may result in probabilities > 100% or < 0%. To address these issues, we re-ran the model transforming efficacy into its logit (${\text{ln}}\left( {\frac{e}{100 - e}} \right)$, where e represents efficacy on a 0 to 100 scale). We selected the final model based on goodness of fit using the Bayesian information criterion (BIC).

The coefficients from mixed logit models do not have intuitive meanings beyond the signs. To increase interpretability, we rescaled them so that the predicted score (called utility, a measure of preference) for the combination of least preferred attributes was zero (assuming the lowest efficacy for a PrEP intervention was a 50% risk reduction) and the combination of most preferred attributes (assuming a maximum PrEP efficacy of 99% risk reduction) was 100. Usual Care was assigned a PrEP efficacy of 1%, since the logit of zero is undefined. We used affine transformations (applying both a linear transformation and a translation, such that both the linear and ratio characteristics of the coefficients are preserved), such that the lowest and highest utility scores calculated from combinations of model parameters were assigned values of 0 and 100, respectively.

Next, we calculated the marginal rate of substitution between attributes with efficacy in the denominator, to answer the question “How much PrEP efficacy is an individual willing to forego in order to realize gains in other attribute levels?” Details of the calculations using the logit transformed efficacy parameter are included in the Supplemental Appendix. The mixed logit model calculates a mean value and the distribution (which we assumed to be normal) for random parameters. To further explore heterogeneity, we calculated and graphed the distribution of individual-level predicted values for selected parameters (on a 0–100 scale). These analyses should be viewed as exploratory.

We conducted three sensitivity analyses. First, we included participants who gave invariant responses. Second, we excluded participants who gave possibly inconsistent responses. We identified these individuals as those who stated that they were “Very Interested” in PrEP on a 5-point Likert scale (maximum score) but selected Usual Care over a PrEP intervention consisting of a daily pill without side effects, 99% efficacy, and no decrease in future treatment options. Third, we performed a similar analysis to the second sensitivity analysis but expanded the definition of inconsistent to include participants who stated they were either “Very Interested” or “Interested” in PrEP.

We used NGene software (version 1.1) for the DCE design and Stata software (version 16.1) for statistical analysis.

### Sample size

Our sample size was pre-determined by the size of the parent survey, which enrolled 400 participants^12^. Of these, 66 were administered the pilot DCE, 31 were administered no DCE, and 303 participants were administered the final version of the DCE.

### Ethics

The study was approved by the Research Ethics Boards of St. Michael’s Hospital, Ryerson University and the University of Toronto. All participants reviewed a written letter of information prior to participation, and survey completion was interpreted as implied informed consent. Participants received a $10 CAD gift card upon survey completion.

### Ethics approval

This study was conducted in line with the principles of the Declaration of Helsinki. The study was approved by the Research Ethics Boards of St. Michael’s Hospital, Ryerson University and the University of Toronto.

### Consent to participate

All participants reviewed a written letter of information prior to participation, and survey completion was interpreted as implied informed consent.

## Results

We recruited 303 participants and excluded one transgender man, 11 who were taking PrEP, and 38 who had invariant responses, leaving 253 participants in the main analysis. The 38 with invariant responses had lower HIRI-MSM risk scores than those in the main analysis, with median (interquartile range, IQR) 8.5 (5, 15) versus 15 (8, 21), *p* < 0.001. Among included participants, median age was 29 (25, 36) years and the median number of sexual partners in the preceding six months was 6 (3, 10) (Table 1). Most participants identified as White (55%) or Asian (17%) and most had a college/undergraduate (51%) or graduate/professional degree (35%). The vast majority were aware of TDF/FTC-based PrEP (92%) and post-exposure prophylaxis (PEP; 77%); a minority (10%) had previously used PEP. Most respondents rated themselves as being at “a little bit of risk” of acquiring HIV in the next year (75%), 22% reported being “more than a little bit” or “very” concerned about their level of HIV risk, and 36% reported ≥ 1 prior bacterial sexually transmitted infection.

**Table 1:** Participant characteristics (N = 253).

Characteristics	n	(%)
Age—median (IQR)	29	(25–36)
**Ethnicity**		
White	140	(55)
Asian	43	(17)
Latino/Hispanic	21	(8)
South Asian	17	(7)
Middle Eastern	12	(5)
Black	10	(4)
Other	10	(4)
**Education**		
High school	35	(14)
College/undergraduate	128	(51)
Graduate/professional	88	(35)
Missing	2	(1)
**Employment**		
Full-time	180	(71)
Part-time	36	(14)
Not employed	37	(15)
Aware of PrEP	232	(92)
**Level of interest in PrEP**		
Very uninterested	7	(3)
Uninterested	30	(12)
Neutral	65	(26)
Interested	67	(26)
Very interested	82	(32)
Missing	2	(1)
Aware of post-exposure prophylaxis	194	(77)
Prior use of post-exposure prophylaxis	25	(10)
Sexual partners in the last 6 months—median (IQR)	6	(3.0 to 10.0)
Condomless receptive anal sex acts in the last 6 months—median (IQR)	0	(0 to 2.0)
HIV risk index^a^—median (IQR)	15	(8 to 21)
HIV risk index ≥ 10	172	(68%)
**Self-perceived risk of acquiring HIV over the next year:**		
No risk	34	(13)
A little bit of risk	190	(75)
More than a little bit (medium) risk	23	(9)
A lot of risk	5	(2)
Missing	1	(0)
**Concerned about current level of HIV risk**		
Not concerned	57	(23)
A little bit concerned	135	(53)
More than a little bit concerned	33	(13)
Very concerned	23	(9)
Missing	5	(2)
**Would take pills before and after sex**		
Strongly disagree	6	(2)
Disagree	16	(6)
Neutral	32	(13)
Agree	85	(34)
Strongly agree	111	(44)
Missing	3	(1)
**Would take a pill every day**		
Strongly disagree	6	(2)
Disagree	29	(11)
Neutral	48	(19)
Agree	78	(31)
Strongly agree	90	(36)
Missing	2	(1)
**Would take PrEP even though it isn’t 100% effective**		
Strongly disagree	11	(4)
Disagree	24	(9)
Neutral	76	(30)
Agree	92	(36)
Strongly agree	47	(19)
Missing	3	(1)
**Prior history of sexually transmitted infections (ever)**		
Gonorrhea	61	(24)
Chlamydia	49	(19)
**Syphilis**		
No	225	(89)
Yes	24	(9)
Missing	4	(2)
Any bacterial sexually transmitted infection	92	(36)

Scale authors proposed that scores of ≥ 10 be used to identify MSM at substantial risk of HIV infection who should be prioritized for PrEP.

^a^The HIV Incidence Risk Index for MSM score is a validated clinimetric scale for quantifying HIV risk in the next 6 months^19^.

Almost all respondents (98%) answered each of the DCE questions. Across all responses, Usual Care was selected 35% of the time. Individuals who were not interested in PrEP were considerably more likely to select Usual Care (62% of responses) than individuals who were very interested (20%, *p* < 0.001 across all response categories, Supplemental Appendix Table S1).

Coefficients from the mixed logit model were generally similar when efficacy was entered as a linear variable (Supplemental Table S2) and when it was entered as the logit of efficacy (Table 2) but the efficacy logit model had a better fit (BIC 4862.3 vs. 4943.9). We used the efficacy logit model for all subsequent analyses. Mc-Fadden’s pseudo-R^2^ for this model was 0.23, which is considered acceptable^17^. The most preferred PrEP formulation was an on-demand pill, while the least preferred option was the rectal gel. The presence of side effects was associated with reduced utility scores for the on-demand pill (*p* = 0.026) and daily pill (*p* = 0.054) but not for rectal formulations or injection. The risk of inducing HIV drug resistance was associated with a significant decrease in utility when this risk was moderate, but not when it was low or zero. Standard deviation (SD) estimates suggested that the most heterogeneity in preferences was associated with the side effect of rectal discomfort (SD = 4.8). Moderate heterogeneity was also observed for Usual Care (i.e., not taking PrEP, SD = 1.6), monthly injection (SD = 1.4), rectal gel (SD = 1.5), nausea with a daily pill (SD = 1.3), and a moderate chance of future decrease in treatment options (SD = 1.4).

**Table 2:** Results of mixed multinomial logit model incorporating efficacy as the logit of efficacy.

	Mean			SD		
	Coefficient	(95% CI)^a^	*P* value	Coefficient	(95% CI)	*P* value
Efficacy (logit)	0.926	(0.810 to 1.041)	< 0.001	N/A		
Usual method	4.588	(3.715 to 5.461)	< 0.001	1.555	(1.103 to 2.007)	< 0.001
**Route and frequency**						
A pill taken every day	0	(Referent)				
A pill taken on-demand with sex	0.966	(0.489 to 1.443)	< 0.001	0.978	(0.524 to 1.433)	< 0.001
An injection taken once a month	0.666	(0.161 to 1.172)	0.010	1.361	(0.887 to 1.835)	< 0.001
A solution inserted into the rectum after sex	− 0.553	(− 1.086 to − 0.019)	0.042	1.534	(0.592 to 2.475)	0.001
**Side effects**						
Nausea with daily pill	− 0.421	(− 0.848 to 0.007)	0.054	1.325	(0.740 to 1.909)	< 0.001
Nausea with on-demand pill	− 0.336	(− 0.633 to − 0.039)	0.026	0.085	(0.289 to 0.459)	0.657
Pain at injection site	0.234	(− 0.121 to 0.590)	0.196	0.191	(− 1.678 to 2.060)	0.841
Rectal Discomfort	− 0.841	(− 2.233 to 0.551)	0.236	4.823	(2.127 to 7.518)	< 0.001
**Risk of decreasing future treatment options (HIV drug resistance)**						
No chance	0	(Referent)				
Low chance	− 0.134	(− 0.402 to 0.135)	0.328	0.503	(− 0.321 to 1.326)	0.232
Moderate chance	− 1.725	(− 2.158 to − 1.292)	< 0.001	1.446	(1.057 to 1.836)	< 0.001

^a^CI denotes confidence interval.

The rescaled coefficients yield scores for combinations of hypothetical PrEP option attributes (Box 1). Thus, an on-demand pill with no side effects, no risk of resistance, and 99% risk reduction efficacy was the most preferred option (utility score = 100), while a rectal gel causing mild rectal discomfort, moderate risk of resistance and 50% efficacy was the least preferred option (utility score = 0). The utility of 41.4 for Usual Care means that any hypothetical PrEP option scoring > 41.4 would be preferred, on average, over not taking PrEP. An excel spreadsheet to calculate utilities is available online (www.optionslab.ca/projects/prep-DCE/).

**Box 1:** Formula for calculating utility scores on a scale from 0 (least preferred combination of attributes) to 100 (most preferred combination of attributes)^a^.

$$\begin{aligned} Utility & = 37.40 + 11.10 \times \ln \left( {\frac{efficacy}{{\left( {100 - efficacy} \right)}}} \right) \\ & \quad + 55.02 \times \left( {Usual\;Method = 1} \right) \\ & \quad + 0 \times \left( {Daily\;pill = 1} \right) \\ & \quad + 11.59 \times \left( {On\;demand\;pill = 1} \right) \\ & \quad + 7.99 \times \left( {Monthly\;injection = 1} \right) \\ & \quad - 6.63 \times \left( {{\text{Re}} ctal\;solution = 1} \right) \\ & \quad - 5.05 \times \left( {Nausea\;with\;daily\;pill = 1} \right) \\ & \quad - 4.03 \times \left( {Nausea\;with\;on\;demand\;pill = 1} \right) \\ & \quad + 2.81 \times \left( {Pain\;at\;injection\;site = 1} \right) \\ & \quad - 10.08 \times \left( {Rectal\;discomfort = 1} \right) \\ & \quad + 0 \times \left( {No\;risk\;of\;resistance = 1} \right) \\ & \quad - 1.60 \times \left( {Low\;risk\;of\;resistance = 1} \right) \\ & \quad - 20.69 \times \left( {Moderate\;risk\;of\;resistance = 1} \right) \\ \end{aligned}$$	

^a^Efficacy = 50% for least preferred combination and 99% for most preferred.

The formula calculates point estimates for individual utility scores based on results of the mixed logit model.

We next used the rescaled coefficients (Box 1) to calculate how much more efficacious PrEP would need to be compared to Usual Care to be preferred (Table 3). An on-demand pill without side effects would be preferred, on average, if it decreased HIV acquisition risk by at least 32.6% more than Usual Care. In contrast, a rectal gel causing mild discomfort would need to decrease HIV acquisition risk by at least 85.6% compared with Usual Care. Because an on-demand pill was the preferred PrEP option, we repeated these calculations with this as the comparator (Table S3). Assuming that an on-demand pill has an efficacy of 86% (estimated efficacy in the intent-to-treat population from the IPERGAY trial^3^), a rectal gel without side effects would have to have an additional efficacy of 10.9% (95% confidence interval [95CI] 9.7% to 12.2%) to be the preferred choice (i.e. efficacy ≥ 96.9% compared to no PrEP). If an on-demand pill had an efficacy of 40% (lower 95% confidence limit from the IPERGAY trial^3^), a daily pill with side effects would need to have an additional efficacy of 34.9% (95CI 26.0 to 43.8%) or at least 74.9% effective compared to no PrEP.

**Table 3:** Minimum efficacy for PrEP options to be preferred over usual care^a^.

PrEP option	Minimum efficacy (95% Confidence Interval) (%)
Daily pill	57.9 (44.1–71.7%)
Daily pill with side effects	68.3 (57.4–79.3%)
On-demand pill	32.6 (21.2–43.9%)
On-demand pill with side effects	41.1 (27.5–54.7%)
Monthly injection	40.1 (27.0–53.2%)
Monthly injection with side effects	34.2 (20.4–47.9%)
Rectal solution	71.3 (60.5–82.1%)
Rectal solution with side effects	85.6 (69.2–100.0%)^b^

^a^Usual care was assumed to have an efficacy of 1%.

^b^Upper 95% confidence limit truncated at 100%.

**Figure 2:**
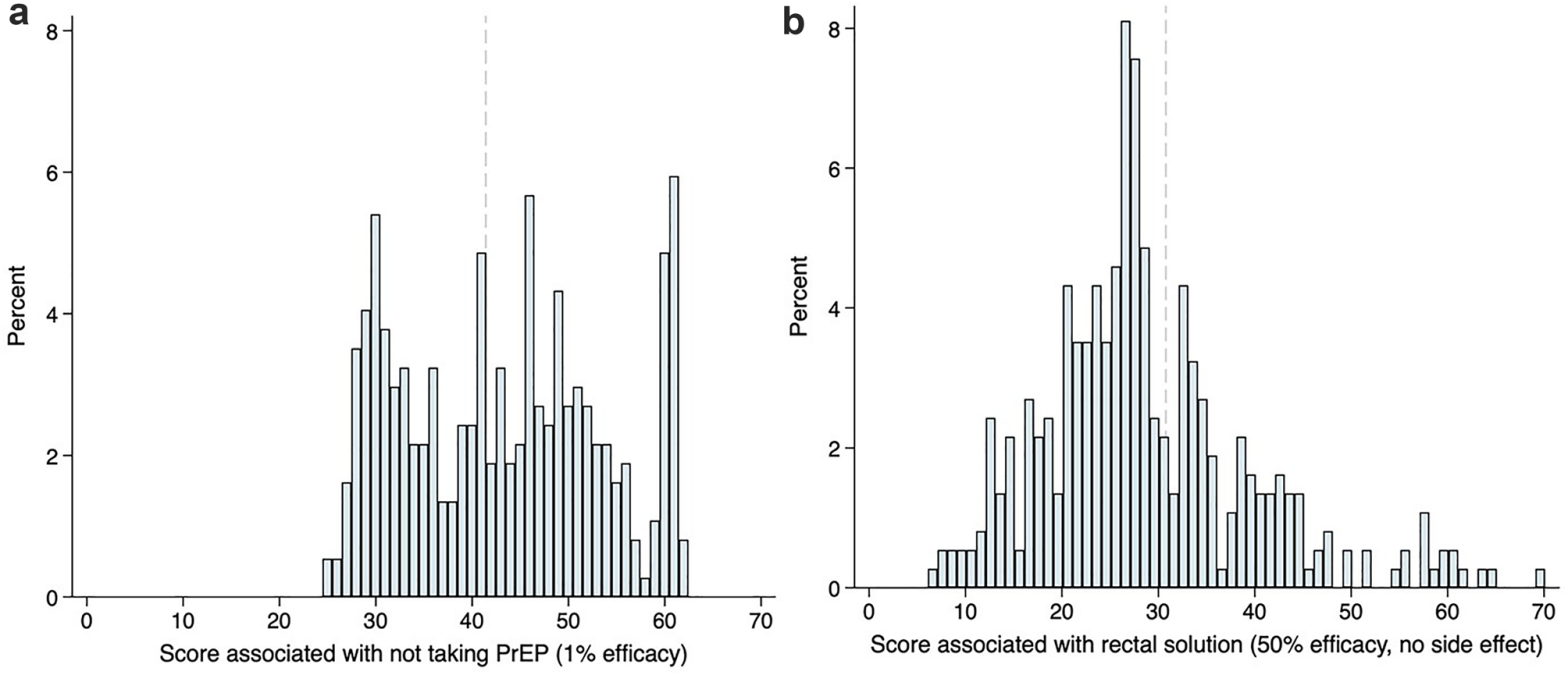
Heterogeneity in utility scores for Usual Care (no PrEP) and for rectal gel as PrEP. Distribution of predicted individual rescaled utility scores for Usual Care (not taking PrEP, panel **A**) and for a rectal gel (panel **B**).

Model results represent mean respondent preferences. To explore heterogeneity in preferences across respondents, we graphed predicted utility scores for the Usual Care (no PrEP) option on the 0–100 scale. The histogram of scores (Fig. 2A) suggests three peaks. About one-third of the sample had low scores for Usual Care (implying high willingness to take PrEP), about 13% had high utility scores, implying an aversion to PrEP, and the remaining participants had mid-range scores, indicating they would consider PrEP under some conditions. A histogram of scores for a rectal gel suggested a unimodal but wide distribution of preferences (Fig. 2B).

## Discussion

We characterized preferences regarding existing and forthcoming PrEP formulations among Toronto gbMSM undergoing anonymous HIV testing in 2016. The least preferred option was a rectal gel that induced mild local discomfort, carried a moderate risk of drug resistance, and conferred only 50% risk reduction, while the most preferred option was an on-demand pill with no side effects, no risk of drug resistance, and 99% HIV prevention efficacy. Importantly, we observed considerable heterogeneity in preferences, suggesting that having a wide range of choices has the potential to motivate some people who would not take PrEP to consider doing so.

On-demand oral TDF/FTC comes close to having the attributes of our participants’ most preferred PrEP formulation. In the IPERGAY trial and open-label extension study, overall efficacy was 86–97%, no participants acquired drug-resistant HIV, and 89–90% of PrEP users experienced no grade 3–4 adverse events^3,4^. The low frequency of side effects with this regimen is further corroborated by the ADAPT trial, in which only 4.5–13.3% of gbMSM in Harlem and Bangkok experienced any neurologic or gastrointestinal side effects with event-driven TDF/FTC PrEP^20^. The low risk of drug resistance with this regimen is supported by data showing that no reverse transcriptase mutations emerged during 28 days of TDF monotherapy in two studies of HIV-positive adults^21,22^. Existing data suggest that other potential oral PrEP regimens such as maraviroc and tenofovir alafenamide/emtricitabine also have few side effects and resistance risk^23–26^, but their viability as on-demand dosing regimens is unknown.

The HPTN 083 trial recently showed that two-monthly injectable long-acting cabotegravir reduced incident HIV infections by 66% compared to daily oral TDF/FTC in gbMSM and transgender women, reaching criteria for statistical superiority^6^. While 80.9% of trial participants reported injection site reactions, most were mild to moderate in severity. Although virologic resistance data from the trial have not yet been reported, our findings suggest that the combination of reported efficacy and tolerability of injectable cabotegravir result in a PrEP option that some individuals may prefer.

Few DCE studies have investigated PrEP-related preferences among gbMSM. In a U.S. study among men recruited in 2015 (N = 554), PrEP modality (daily pill, on-demand pill or monthly injection) was the second most important driver of preferences for a PrEP program after costs, followed by the type of adherence support, prescription practices, and dispensing venue^27^. In contrast to our sample, the scenario that maximized willingness to take PrEP in that study involved a daily as opposed to on-demand pill, although subsets of participants favoured an injectable option. In a South African study regarding long-acting PrEP formulations among 807 participants including 190 MSM, dosing frequency was more important than formulation, access, pain, insertion site as a driver of preferences, although MSM also exhibited a strong preference for injections over implantable formulations^28^. A DCE among gbMSM and transgender women in Thailand regarding rectal microbicides found greater appeal for a gel versus a suppository, and for intermittent versus daily dosing^29^. Not surprisingly, and consistent with our observations, DCE studies that included HIV prevention efficacy as an attribute generally found that it was the primary driver of user preferences^29–31^; this was further reinforced in a recent DCE among MSM in India^32^. Higher efficacy was also the primary determinant of acceptability in studies regarding HIV vaccines^33,34^. The heterogeneity of preferences in these studies highlights the importance of developing a variety of PrEP formulations. Fortunately, there remains a rich pipeline of products in development for various populations, including rectal suppositories^35^, vaginal rings combining dapivirine with hormonal contraceptives^36^, and subdermal implants containing a variety of existing and forthcoming antiretroviral agents^37^.

A relatively novel approach in our study was to use the marginal rate of substitution to quantify the trade-off in HIV prevention efficacy that participants were willing to accept in favour of desirable product attributes. This approach is analogous to quantifying the willingness to pay for a product using a contingent valuation approach, but uses changes in risk of HIV acquisition as the numerator, rather than currency units. A previous study of HIV prevention options among men and women in South Africa used a similar approach^31^. These findings provide a framework for decision-making about products with statistically significant but clinically suboptimal prevention efficacy (or effectiveness), and whether such products warrant regulatory approval because of their potential appeal to specific groups of users. Such reasoning underlies current efforts to obtain regulatory approval for the dapivirine vaginal ring as PrEP, whose modest HIV risk reduction of ~ 30% in clinical trials is ‘worth the trade-off’ because of the ring’s other appealing attributes including minimal side effects, ease of use and potential to be concealed from sexual partners^38,39^. The marginal rate of substitution in HIV prevention efficacy could also be used in the design of clinical trials. For example, if a novel PrEP product offering a desirable attribute were to be compared against an existing standard of care, our results could provide a patient-centred approach to defining the non-inferiority threshold for HIV prevention efficacy.

Our study has imitations that warrant consideration. First, we made several simplifying assumptions, particularly regarding side effects, which we assumed were static, rather than dynamic, and universally mild. Thus, we may have over-estimated the importance of the gastrointestinal side effects of daily TDF/FTC-based PrEP, which typically subside over time. Second, we only considered four attributes, and the levels that we selected may not match those of products that reach the regulatory approval stage. For instance, we suggested that injectable PrEP would be administered monthly, based on prior cabotegravir data^40,41^, however the dose shown to be superior to daily oral TDF/FTC was every 8 weeks, suggesting that injectable PrEP may be favoured even more than we estimated. We also did not assess other emerging PrEP options such as implants. Third, we did not assess monetary willingness to pay. This decision was deliberate, to reflect the Ontario healthcare context in which medically necessary drugs are at least partially (and sometimes fully) covered under the public drug formulary. Fourth, any DCE is subject to hypothetical bias, meaning that the opinions expressed by survey respondents may not match the decisions they would actually make under real-world conditions. Fifth, while the ethnoracial diversity of our study sample roughly corresponds to that of metropolitan Toronto, we had a relatively lower proportion of Black participants (4% of our sample versus 9% of the population)^42^, and non-English speakers were excluded. A modest number of participants with invariant responses were also excluded, although these participants tended to have lower rather than higher HIV risk. Sixth, we assumed that all individuals interpreted “usual care” similarly, although there may be heterogeneity in HIV prevention practices among participants. Finally, preferences regarding PrEP may be dependent on social context. We conducted our study in Toronto, Canada during the summer of 2016, when knowledge about TDF/FTC-based PrEP was rapidly rising^12^, but access remained limited, such that generalizing to current populations with broader PrEP availability may not be appropriate. As the social acceptability of PrEP and knowledge about forthcoming PrEP options increase, attitudes towards novel formulations may change. Preferences may also vary based on individual patient characteristics such as underlying level of HIV risk, and our study design did not account for such potential differences.

While these issues may limit the generalizability of our model findings to MSM in other settings, the underlying message about the importance of multiple PrEP options is broadly applicable to other populations. Our findings should not be directly applied to non-MSM populations, because differences in sexual practices (eg decreased relevance of anal sex) and in the menu of PrEP options in development (eg agents designed for the female genital tract) may differ substantially.

## Conclusions

Having a menu of PrEP options is important for appealing to a range of at-risk MSM in Toronto. Characterizing these preferences may also inform trial design as well as strategies to maximize uptake.

## Supplementary Information

Supplementary Information.

## Data Availability

Study data are available from the authors upon request.
