# Supplementary material for: Preferences regarding emerging HIV prevention technologies among Toronto men who have sex with men: a discrete choice experiment
Source: Sci Rep. 2021 Nov 15;11:22252. doi: 10.1038/s41598-021-01634-3 (PMC8592986; doi:10.1038/s41598-021-01634-3)
Supplement: Supplementary file 1 — Supplementary Information. [file 41598_2021_1634_MOESM1_ESM.docx]

**Marginal Rate of Substitution with a Logit-Transformed Variable**

The marginal rate of substitution is typically calculated as the amount of a continuous variable, *c*, that an average individual would need to acquire (or forego), in order to compensate for a change in level of a categorical variable, *X*, say from level *m* to level *n.* It is typically calculated as

$${mrs}_{X_{nm}}=- \frac{U_{X_{m}}-U_{X_{n}}}{U_{C}}$$

Where *mrs* represents the marginal rate of substitution, $U_{X_{m}}$and $U_{X_{n}}$ represent the utility (coefficients) associated with attribute levels *m* and *n* of attribute *X*, and $U_{C}$ represents the utility of *C*, which is often a cost. When level *n* of attribute *X* is the reference category, $U_{X_{n}}$=0 and the marginal rate of substitution is equal to the ratio of the two utilities, which are coefficients of the equations. The utility of *C* is given by $\beta_{c} c$, where $\beta_{c}$ represents the beta coefficient associated with cost and *c* represents the amount paid. Typically, we are interested in the change in one unit of cost so *c* = 1.

The derivation of this formula comes from the utility score, where the total utility, *U*, is the sum of the utilities associated with each attribute/level combination. If all other attributes are set to constant values, it is possible to find two values of *c* whereby

$$Ux_{m}+{U_{C}}_{2}=Ux_{b}+U_{C_{1}}$$

Regrouping and substituting coefficients yields

$$\beta x_{m}-\beta x_{b}=(\beta_{c} c_{1}- {\beta_{c} c}_{2})$$

Thus, the dollar value of this utility change = $c_{2}-c_{1}=$ $-\frac{\beta x_{m}-\beta x_{b}}{\beta_{C}}$, which is the marginal rate of substitution formula.

The marginal rate of substitution is for a change in probability is conceptually more complicated. Mathematically, probabilities are bounded by 0 and 1. Psychologically, people frequently do not treat probabilities as linear.

One solution is to use a transformation of the probability scale (Figure S1). The logit transformation is given by the formula

$$logit\left( p \right)=ln(\frac{p}{1-p})$$

Graphically, the logit is sigmoid in shape. It is undefined at 0 and 1; at low and high probabilities, the function increases rapidly, which may be interpreted as a strong aversion to low (uncertain) probabilities and a strong preference for high (certain) probabilities.

**Figure S1. Logit transformation of the probability scale**

Figure legend: Graphically, the logit is sigmoid in shape. It is undefined at 0 and 1; at low and high probabilities, the function increases rapidly, which may be interpreted as a strong aversion to low (uncertain) probabilities and a strong preference for high (certain) probabilities.

We refer to the coefficient associated with a unit change in the logit as $\beta_{l}.$Substituting the logit of probabilities for costs yields the formula

$$\beta x_{m}-\beta x_{b}=\beta_{l} {logit(p}_{1})- {\beta_{l} logit(p}_{2})$$

Thus,

${logit(p}_{2})-{logit(p}_{1})=$ $-\frac{\beta x_{m}-\beta x_{b}}{\beta_{l}}$

However, it is convenient to express this as a difference in probability, not in the logit of probability. To put these estimates back on a probability scale, it is necessary to select a reference level for $p_{1}$ and conduct sensitivity analyses at different levels.

To formula to derive the probability, $p_{2}$, at which the utility is equivalent is

$$p_{2}=invlogit\left\lfloor logit\left( p_{1} \right)-\frac{{\beta_{X}}_{m}-\beta_{X_{b}}}{\beta_{l}} \right\rfloor$$

where

$$invlogit(p)=\frac{e^{p}}{1+e^{p}}$$

The marginal rate of substitution for probability therefore varies across different levels of probability

$${mrs}_{X_{mb}}(p_{1})= invlogit\left\lfloor logit\left( p_{1} \right)-\frac{{\beta_{X}}_{m}-\beta_{X_{b}}}{\beta_{l}} \right\rfloor-p_{1}$$

Table S1 Interest in PrEP and Discrete Choice Experiment Responses

| **Interest in PrEP** | **PrEP Option 1** | | **PrEP Option2** | | **Usual Care** | |
| --- | --- | --- | --- | --- | --- | --- |
|  | **N** | **(%)** | **N** | **(%)** | **N** | **(%)** |
| Very uninterested | 45 | (17.9) | 51 | (20.2) | 156 | (61.9) |
| Uninterested | 258 | (23.9) | 255 | (23.6) | 567 | (52.5) |
| Neutral | 579 | (24.8) | 600 | (25.7) | 1158 | (49.6) |
| Interested | 906 | (38.1) | 819 | (34.4) | 654 | (27.5) |
| Very interested | 1194 | (40.8) | 1149 | (39.2) | 585 | (20.0) |
| **Total** | **2982** | **(33.2)** | **2874** | **(32.0)** | **3120** | **(34.8)** |

PrEP denotes Pre-Exposure Prophylaxis

Table S2 Mixed Logit Model Results with Efficacy as a Linear Variable

|  | **Mean** | | |  | **Standard Deviation** | | |
| --- | --- | --- | --- | --- | --- | --- | --- |
|  | **Coefficient** | **(95% Conf. Int.)** | **p-value** |  | **Coefficient** | **(95% Conf. Int.)** | **p-value** |
| Efficacy (logit) | 0.076 | (0.065 to 0.086) | <0.001 |  | N/A |  |  |
| Usual method | 4.435 | (3.587 to 5.283) | <0.001 |  | 1.540 | (0.395 to 2.686) | 0.008 |
| Route and Frequency |  |  |  |  |  |  |  |
| A pill taken every day | 0 | (Referent) |  |  |  |  |  |
| A pill taken on-demand with sex | 0.737 | (0.277 to 1.197) | 0.002 |  | 1.310 | (0.765 to 1.856) | <0.001 |
| An injection taken once a month | -0.370 | (-0.784 to 0.043) | 0.079 |  | 1.240 | (0.801 to 1.678) | <0.001 |
| A solution inserted into the rectum after sex | -1.010 | (-1.511 to -0.509) | <0.001 |  | 1.506 | (0.762 to 2.250) | <0.001 |
| Side Effects |  |  |  |  |  |  |  |
| Nausea with daily pill | -0.567 | (-0.964 to -0.170) | 0.005 |  | 1.155 | (0.491 to 1.819) | <0.001 |
| Nausea with on-demand pill | -0.907 | (-1.203 to -0.611) | <0.001 |  | 0.012 | (-0.547 to 0.570) | 0.967 |
| Pain at injection site | 0.714 | (0.378 to 1.049) | <0.001 |  | -0.097 | (-0.547 to 0.354) | 0.674 |
| Rectal Discomfort | 0.541 | (-0.513 to 1.594) | 0.314 |  | 5.195 | (3.424 to 6.965) | <0.001 |
| Risk of decreasing future treatment options (HIV drug resistance) |  |  |  |  |  |  |  |
| No chance | 0 | (Referent) |  |  |  |  |  |
| Low chance | -0.013 | (-0.283 to 0.256) | 0.923 |  | 0.429 | (-0.172 to 1.030) | 0.162 |
| Moderate chance | -1.051 | (-1.450 to -0.652) | <0.001 |  | 1.429 | (1.027 to 1.831) | <0.001 |

Table S3 Minimum Efficacy for PrEP Options to be Preferred over On-Demand Pill

| **Attributes** | **Coefficient** | **(95% Conf. Int.)** | **p-value** |
| --- | --- | --- | --- |
| **On-demand pill efficacy of 50%** |  |  |  |
| Daily pill | 24.0% | ( 14.6% to 33.3%) | <0.001 |
| Daily pill with side effects | 31.7% | ( 24.7% to 38.8%) | <0.001 |
| On-demand pill with side effects | 9.0% | ( 1.1% to 16.9%) | 0.026 |
| Monthly injection | 8.0% | (- 3.6% to 19.6%) | 0.174 |
| Monthly injection with side effects | 1.8% | (- 11.7% to 15.2%) | 0.797 |
| Rectal solution | 33.8% | ( 27.9% to 39.6%) | <0.001 |
| Rectal solution with side effects | 42.8% | ( 32.8% to 52.7%) | <0.001 |
| **On-demand pill efficacy of 70%** |  |  |  |
| Daily pill | 16.9% | ( 11.4% to 22.4%) | <0.001 |
| Daily pill with side effects | 21.3% | ( 17.5% to 25.0%) | <0.001 |
| On-demand pill with side effects | 7.0% | ( 1.3% to 12.8%) | 0.017 |
| Monthly injection | 6.3% | (- 2.3% to 14.9%) | 0.148 |
| Monthly injection with side effects | 1.5% | (- 9.5% to 12.5%) | 0.794 |
| Rectal solution | 22.3% | ( 19.3% to 25.4%) | <0.001 |
| Rectal solution with side effects | 26.8% | ( 22.1% to 31.4%) | <0.001 |
| **On-demand pill efficacy of 90%** |  |  |  |
| Daily pill | 6.2% | ( 4.5% to 8.0%) | <0.001 |
| Daily pill with side effects | 7.6% | ( 6.5% to 8.7%) | <0.001 |
| On-demand pill with side effects | 2.8% | ( 0.7% to 5.0%) | 0.011 |
| Monthly injection | 2.6% | (- 0.7% to 5.8%) | 0.125 |
| Monthly injection with side effects | 0.6% | (- 4.0% to 5.2%) | 0.792 |
| Rectal solution | 7.9% | ( 7.0% to 8.8%) | <0.001 |
| Rectal solution with side effects | 9.1% | ( 7.9% to 10.4%) | <0.001 |
